# Screening, Purification and Characterization of Anionic Antimicrobial Proteins from *Foeniculum Vulgare*

**DOI:** 10.3390/molecules22040602

**Published:** 2017-04-08

**Authors:** Raid Al Akeel, Ayesha Mateen, Rabbani Syed, Abdullah A. Alyousef, Mohammed Rafi Shaik

**Affiliations:** 1 Department of Clinical Laboratory Sciences, College of Applied Medical Sciences, King Saud University, Riyadh 11451, Saudi Arabia; raalakeel@ksu.edu.sa (R.A.A.); ayeshamateen@gmail.com (A.M.); abalyousef@ksu.edu.sa (A.A.A.); 2 Department of Chemistry, College of Science, King Saud University, P.O. Box 2455, Riyadh 11451, Saudi Arabia

**Keywords:** *Foeniculum vulgare*, ion exchange chromatography, antimicrobial proteins

## Abstract

*Foeniculum vulgare* Mill., commonly called fennel, is a medicinal plant belonging to the Umbelliferae (Apiaceae) family, and is used in traditional medicine. Antibacterial peptides were isolated using sodium phosphate citrate buffer and, for extraction, cetyltrimethyl ammonium bromide (CTAB) buffer with pH 6, have been employed and antimicrobial activity tested against four reference strains. The extracted protein was subjected to 3 kDa dialysis and separation was carried out by DEAE-ion exchange chromatography and further proteins were identified by 2D gel electrophoresis. The results of *Foeniculum vulgare* elutes obtained from DEAE-ion exchange chromatography were tested for antibacterial activity. Elute 3 shows the highest antibacterial activity on *Pseudomonas aeruginosa* with a diameter of a zone of inhibition of 16 mm and IC_50_ value 25.02 (mcg/mL). Based on the findings of the wide usage in treatment of various ailments and day-to-day life, *Foeniculum vulgare* seeds were used in the present research and have shown promising antibacterial effects, which requires further proteomic research to authenticate the role of the anticipated proteins.

## 1. Introduction

Voluminous plants and plant extracts have a wide variety of resistance mechanisms to chemical, physical, and biological strains, like heavy metals, cold, drought, pathogens and contaminant attacks from bacteria, fungi, and viruses. Several plants exhibited complete genes, allied together for assimilated control over the infections from numerous types of pathogens [1]. Typically, the resistance control is accomplished by the discharge of secondary metabolites, such as phytoalexins, polyphenolics, and tannins, and the generation of pathogenesis-related proteins. In the early 1970s pathogenesis-associated proteins were primarily revealed from the leaves of tobacco. In response to the infections of tobacco mosaic virus were later well-defined as the induced proteins that are discharged through the pathogenic attacks [2].

Antimicrobial peptides are pervasive and the established host resistances, in contrast to pathogens and pests in different organisms, vary from microorganisms to animals [3]. Antimicrobial peptides occur in various molecular arrangements, while most of them are linear peptides from plants, insects, and animals. However, the bacteria harvest polycyclic peptides, for instance, lantibiotics, and complete principal forms of life harvest round peptides, which comprise bacteriocins from bacteria, theta-defensins from animals, and cyclotides from plants [4,5,6]. The preponderance of antimicrobial peptides from plants are Cys-rich [7]. Generally, numerous mutual features of plant antimicrobial peptides share with those from insects, microorganisms, and animals. They comprise characteristics, like their molecular arrangements. These features are well-signified by two plant antimicrobial peptide families, thionins and plant defensins. For example, knottin-type peptides inhibit enzymes, such as, proteases; hevein-like peptides bind chitins; and lipid transference proteins bind lipids to interrupt microbial permeation into cell membranes.

*Foeniculum vulgare* Mill., commonly called fennel, is a therapeutic plant belonging to the Umbelliferae (Apiaceae) family, is widely used in traditional medicine. This is one the most important plants, which has several pharmacological properties, both in vivo and in vitro, including anti-microbial, anti-viral, anti-inflammatory, anti-mutagenic activities, etc. [8]. Based on scientific evaluation and its use in traditional medicine, *Foeniculum vulgare* emerged as a good source of medicinal products for research, proving noteworthy in the field of pharmaceutical biology, as well as in the research and development for new drugs. Previous study showed that extract from *Foeniculum vulgare* has significant anti-bacterial activity against some tested foodborne pathogens [9]. There are many such authenticated studies where authors tested different parts of this plant, with promising results. Plant seeds possess antimicrobial proteins [10,11], defensins [12,13], thionins [14], lipid transfer proteins [15,16], 2S albumins [17,18], and ribosome-inactivating proteins [19,20,21]. Some of the research studies have confirmed that these proteins that show antimicrobial activity may be employed to generate pathogen resistance in transgenic strains [17,22].

Identification and characterization of proteins is an essential step to understand the key roles of proteins in the cell. In recent times, proteomic analysis has emerged as a successful technology to identify and characterize the loop link proteins that will merge the gap between proteomics and pharmacology. In our study we adopted the gel electrophoresis technique to separate proteins by 2DE, followed by protein identification by mass spectrometry, which is a widely used approach in proteomics.

## 2. Results and Discussion

The protein extract after extracting in sodium acetate buffer, pH-6.5, were purified by dialysis, and the same extract was concentrated and subjected for antibacterial testing against four standard pathogenic bacterial strains found to cause foodborne illness and spoilage of food and herbal drugs. The bacterial strains used in the study were *Escherichia Coli* (*E. coli*), *Pseudomonas aeruginosa* (*P. aeruginosa*), *Staphylococcus aureus* (*S. aureus*), and *Proteus vulgaris* (*P. vulgaris*), and compared with the standard antibiotics ciprofloxacin (25 mcg/mL) and chloramphenicol (100 mcg/mL).

### 2.1. Comparison of Protein Concentration Extracted in Different Buffers after Dialysis

The result of the study shown in Table 1 reveals that different buffers and pH vary in the concentration of protein extracts. Seeds of *Foeniculum vulgare* Mill., showed low concentration of protein, at 80 μg/mL, when extracted in sodium phosphate citrate buffer (pH-7.2) and CTAB buffer (pH 6.0), and exhibited the highest concentration of protein, at 140 μg/mL, with sodium acetate buffer (pH-6.5).

### 2.2. Antibacterial Activity of Sodium Acetate Buffer pH-6.0 Extracts after Dialysis

**Figure 1:**
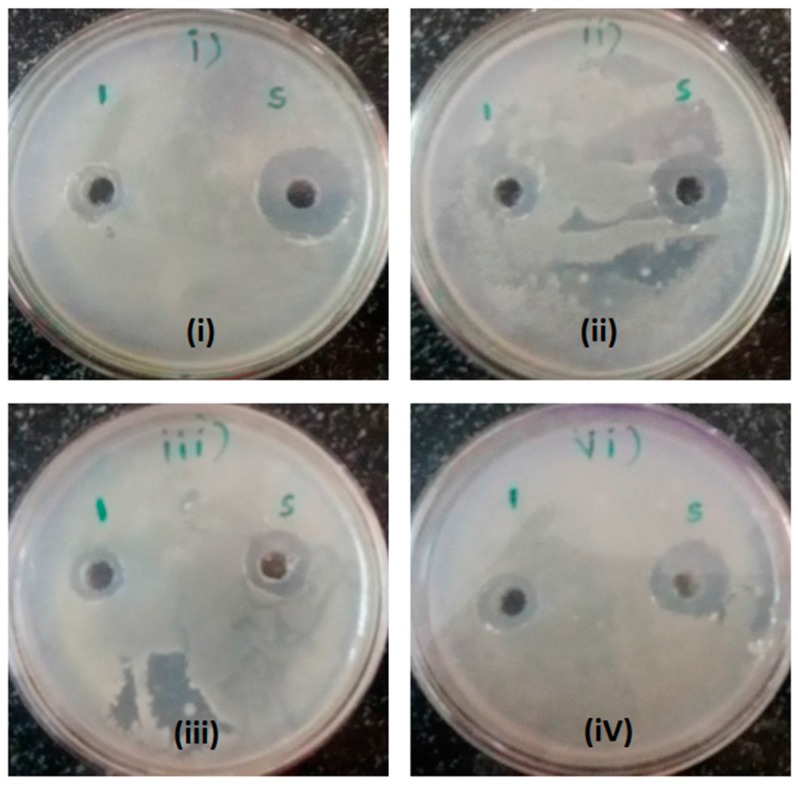
The photograph shows the antimicrobial activity of *Foeniculum vulgare* extracted in sodium acetate buffer at a pH of 6.5 after dialysis. In this figure, ‘1’ indicates protein from *Foeniculum vulgare* and ‘S’ indicates standard antibiotic-Ciprofloxacin (100 mcg/mL). (**i**) *S. aureus*; (**ii**) *E. coli*; (**iii**) *P. aeruginosa*; and (**iv**) *P. vulgaris.*

Antibacterial activity results shown in Table 2 and Figure 1 were obtained from sodium phosphate citrate buffer and CTAB buffer extracts from *Foeniculum vulgare* Mill. (S1) were found to exhibit nil activity on all of the bacterial strains used, so this plant’s seeds were again extracted in sodium acetate buffer, pH-6.5, to obtain a sensitivity pattern on the bacterial strains. *Pseudomonas aeruginosa* exhibited very good sensitivity when compared with the other bacterial strains with a diameter of the zone of inhibition 12.5 mm, whereas *Staphylococcus aureus* and *Proteus vulgaris* showed similar zones of inhibition of 12 mm. *E. coli*, *Pseudomonas aeruginosa*, and *Proteus vulgaris* revealed good zone of inhibition patterns of 11 mm, 12.5 mm, and 12 mm, when compared with the standard antibiotic Chloramphenicol (25 mcg/mL), which is only 8 mm.

### 2.3. Protein Concentration of Ion Exchange Chromatography Elutes

The crude protein extracts from *Foeniculum vulgare* Mill. extracted in sodium acetate buffer at a pH of 6.5 after dialysis were further purified using the DEAE-ion exchange chromatography technique to isolate and characterize the antibacterial proteins present in the extract. The crude protein extract after dialysis was subjected to ion exchange chromatography, four elutes were obtained, and the concentration was found using the Lowry method. The protein concentration was found in ranges from 100–120 mcg/mL, the highest concentration was seen in elute 1 and 2 with 120 mcg/mL, and the least was found in elute 4, with 100 mcg/mL, results shown in the Table 3.

### 2.4. Antibacterial Activity

**Figure 2:**
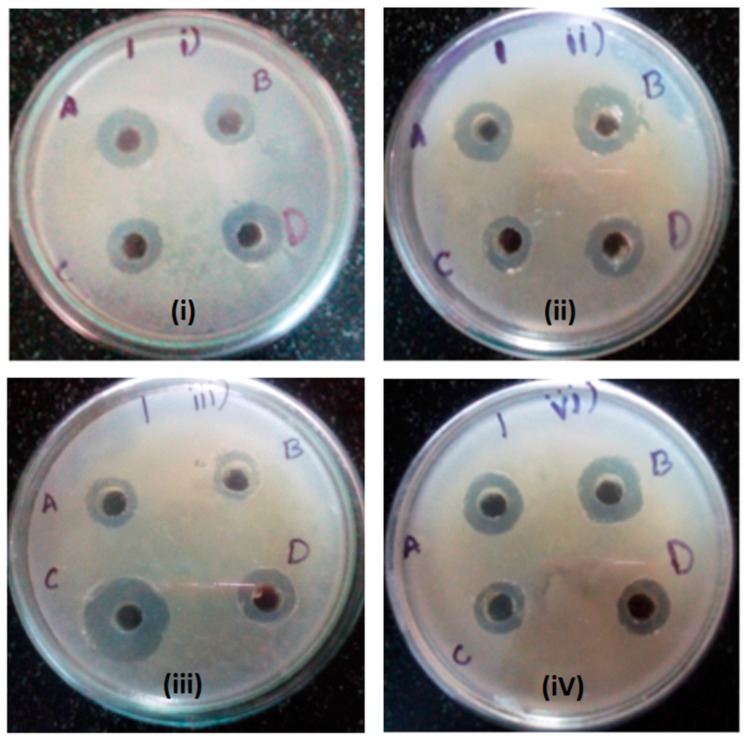
The diameter of the zone of inhibition of ion exchange chromatography elutes from *Foeniculum vulgare*. *S. aureus* (**i**), *E. coli* (**ii**), *P. aeruginosa* (**iii**), and *P. vulgaris* (**iv**). A, B, C, and D represent elutes 1, 2, 3, and 4, respectively.

The results of *Foeniculum vulgare* elutes obtained after ion exchange chromatography were tested for antibacterial activity as shown in Table 4 and Figure 2. Elute 3 shows the highest antibacterial activity on *P. aeruginosa* with a diameter of the zone of inhibition of 16 mm and an IC_50_ value of 25.02 mcg/mL, when compared with the standard antibiotic Chloramphenicol at 100 mcg/mL which is only 12 mm and has an IC_50_ value of 14.634 mcg/mL. Elute 2 shows a good zone of inhibition on *Proteus vulgaris* with a diameter of the zone of inhibition of 13 mm and an IC_50_ value of 57.83 mcg/mL, and the lowest zone of inhibition was seen on *P. aeruginosa* with a zone of inhibition of 4 mm and an IC_50_ value of 68.33 mcg/mL. *S. aureus* was found to shown good sensitivity toward elute 4 with a diameter of the zone of inhibition of 12 mm and an IC_50_ value of 20.8 mcg/m) and, with elute 1, a diameter of the zone of inhibition of 10 mm and an IC_50_ value 27.64 mcg/mL. *E. coli* exhibited good sensitivity towards elute 1 with a zone of inhibition at 13 mm and IC_50_ value of 67.56 mcg/mL.

The analysis was performed using SAS for Windows version 9.2 (SAS Institute Inc., Cary, NC, USA). One-way ANOVA was used to compare the IC_50_ of different elutes on four bacterial strains used in the study. Among the tested strains, *S. aureus* and *P. vulgaris* showed the highest significant inhibition in comparison to standard cipro (*p* = 0.02) whereas there is no significance observed in the IC_50_ in *E. coli* and *P. aeruginosa* compared to standard cipro, results shown in Table 5.

### 2.5. 2-D Gel Electrophoresis Results

**Figure 3:**
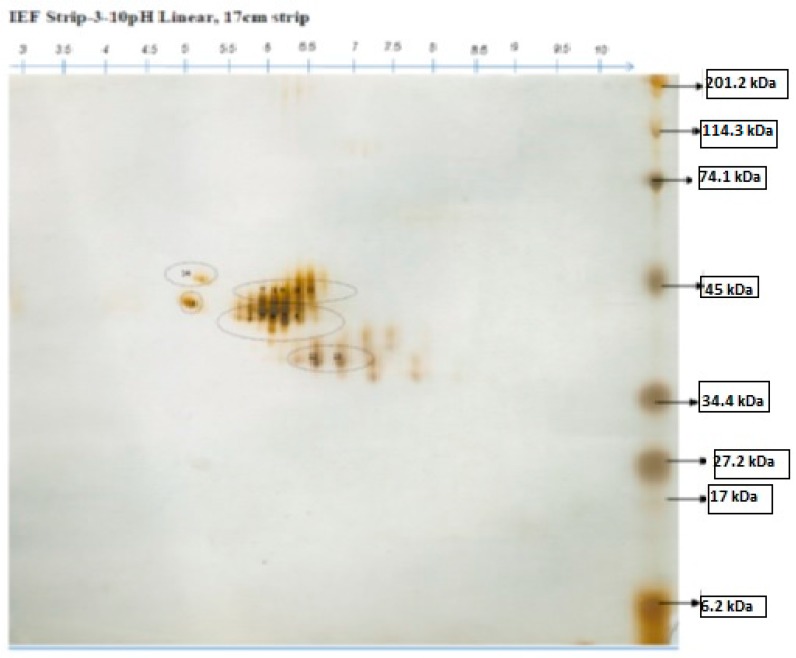
The protein spots of elute 2 obtained after ion exchange chromatography. Marker used: Biorad Prestained SDS-PAGE Standard, Broad Range. IEF strip pH 3–10 linear, 17 cm.

The purified proteins from *Foeniculum vulgare* seeds after anion exchange chromatography were subjected to 2-DE. Out of four elutes obtained from anion exchange chromatography, only elute 2 was used for 2-DE analysis. The elute 2 protein sample extracted from *Foeniculum vulgare* seeds was analyzed to investigate the proteins present in the sample based on the molecular weight, using HP Scanjet G4010 proteomic techniques. The analysis of 2D gel of *Foeniculum vulgare* showed reproducible protein spots, most of which were distributed near the center of the gels, between pI values 5–8 and molecular weight of 34.4 kDa and 48 kDa (Figure 3).

Antibiotic resistance is one the most dangerous global problems today, which makes antimicrobial proteins/peptides the agents of a substitute for cures of pathogenic diseases [23]. In this study, we have extracted 3 kDa antimicrobial peptides from *Foeniculum vulgare* Mill. seeds using buffers and the specific proteins of interest were purified with a dialysis technique using 3 kDa dialysis tubing. Conversely, further separation was done using DEAE-ion exchange chromatography and four elutes were subjected to antimicrobial activity, out of which elute 2 has promising results, which was then characterized by using 2D gel electrophoresis. Much expected optimistic results were obtained against tested pathogenic bacteria, where they exhibited antibacterial activity in comparison to the standard cipro (100 mcg). Past studies have demonstrated that asymptomatic bacteriuria posed more serious hazards in pregnant women [24], so bacterial resistance is inciting a resurgence in research of the antimicrobial activities of herbs against resistant strains [25,26].

In the present research work on anion exchange resin diethyl amino ethyl cellulose (DEAE) was used, which is positively-charged to exchange negatively-charged protein. DEAE-ion exchange chromatography is one of the promising techniques used to separate proteins based on their surface ionic charge using resins that are altered with either negatively-charged or positively-charged biochemical groups [27]. As in this study, anion exchange resin DEAE is being used, as proteins purified by ion exchange chromatography may contain a negative charge.

Proteomics is a handy technique used in the characterization of various proteins [28]. Additionally, 2-DE polyacrylamide gel electrophoresis is a potent technique and, as such, can be adopted to separate and determine protein mixtures of thousands of specific components [29], is based on the gel size, is capable of determining more than 5000 proteins at once, and can detect with less than 1 mg of sample per spot. In our research work, 2-D PAGE is used for the characterization of anionic proteins from elute 2 of the ion-exchange chromatography sample of *Foeniculum vulgare* seed extract, and most of the protein spots were found to be between pI values 5–8 and molecular weights of 34.4 kDa and 48 kDa on the 2D gel, which was found to be higher in molecular weight and possess antibacterial activity from being tested on the four bacterial strains. Previous study identified napins, a class of proteins from 2S albumin, which were isolated from plants and that can be easily extracted from seeds [30] which are composed of 10 kDa and 4.5 kDa subunits [31]. Furthermore the present study has provided more evidence that the high mass protein isolated from the Gram-negative plant pathogen, and also more researchers’ stated literature reports on carotovoricin [32] and colicins [33,34,35], authenticating our present research study.

## 3. Experimental Section

### 3.1. Materials

The *Foeniculum vulgare* Mill. seeds were procured from a local market in Riyadh, Saudi Arabia. Before adopting the protocol, the plant seeds were identified by Dr. Ali S. Alqahtani, Pharmacognosy Department, King Saud University, Riyadh, Saudi Arabia. Ethical approval was obtained from the research ethics committee (CAMS-153-36/37) from the college of Applied Medical Sciences, King Saud University, Riyadh, Saudi Arabia.

### 3.2. Preparation of Plant Seeds Extract

The *Foeniculum vulgare* Mill. seeds were cleaned using sterile water to remove any debris left over on the seeds and dried in a biological safety cabin to maintain the sterile condition. Later, fine powder was prepared from the seeds under aseptic conditions and the extract was soaked in sodium phosphate buffer (pH-6.5) at 30 °C and left for overnight incubation. The next day, extracts were filtered with Whattmann filter paper No. 1 and subjected to 80% ammonium sulfate saturation. The collected saturated material was then separated by dialysis using a 3 kDa cutoff dialysis tubing (Sigma Aldrich, St. Louis, MO, USA) and the protein’ concentration was further estimated by spectroscopic analysis at a wavelength of 280 nm.

### 3.3. DEAE-Ion Exchange Chromatography

The chromatography column was prepared using a DEAE cellulose bed of 1 cm thickness, washed with ethanol before use, 20 mM Tris-Hcl for the 250 mL required volume was prepared, and pH was attuned to 8.5 using NaOH. Bed preparation: counter ions (salt gradient) of 40 mL of 25 mM NaCl in 50 mM tris (pH-7.2) and 0.3% of DEAE were applied to the column. The elutes were run for 4 h and 2 mL was collected. Each column used a flash chromatography column from Sigma Aldrich, St. Louis, (MO, USA), and the eluting buffer was sodium phosphate citrate buffer (pH-7.2). Elute preparation: four elutes were prepared in test tubes for each dialyzed sample. After dialysis, the samples were poured into the column without disturbing the bed, and left for 20 min to settle. The first eluting buffer, i.e., sodium phosphate citrate buffer (pH-7.2), was loaded into the column and was used to elute the sample. Thed column was allowed to settle for 15–20 min. The flow rate is adjusted to 1 mL/min and the elute was collected in test tubes at the bottom.

### 3.4. Bacterial Strains

Bacterial strains used for the study were *E. coli*, *P. aeruginosa S. aureus*, and *P. vulgaris*, and were obtained from department of laboratory sciences, College of Applied Medical Sciences, Riyadh, Saudi Arabia.

### 3.5. Culture Medium and Inoculum Preparation

Antibacterial activity of this crude extract was tested against these four strains in Hi-media. Culture slants were prepared by taking a loop full of pure culture and dispensing into 10 mL nutrient broth from four strains in a sterilized laminar flow and incubated for two days in the incubator. The final concentration of each active culture was maintain at 108 cfu/mL.

### 3.6. Agar Well Diffusion Assay

The antibacterial activity of the crude protein extracts was determined by agar well diffusion assay [36]. Pure cultures were dispersed on agar plates using sterile cotton swabs. Next, using a sterile borer, wells were created on the agar plates. Crude protein extracts (100 µL) filled the wells, with antibiotic and buffer as negative controls, and incubated for a day in an incubator. All of the plates were conducted in triplicate to ensure accurate results [37] and the zone of inhibition was measured in millimeters.

### 3.7. Determination of the Minimal Inhibitory Concentration (MIC)

The protein extracts of different plant seeds were tested for antibacterial activity against four strains by the micro-dilution method [38], with some alterations and suggestions by NCCLS (NCCLS, 2001). Different concentrations of extracts were loaded in 96-well plates to check the antibacterial activity of the extracted proteins. The suspension of culture with 10^8^ cfu/mL concentration added to each well and made a final volume to 200 μL by adding LB broth. Ten microliters of MTT (5 mg/mL) was added to all of the wells after incubation at ±4 °C for 18 h and the readings were taken at 530 nm using an ELISA reader (Tecan, Männedorf, Switzerland). The minimum concentration of protein that showed total inhibition was considered as its MIC minimum zone of inhibition. As control experiments, Chloramphenicol (25 mcg) and Ciprofloxacin (100 mcg) were taken as positive controls, and sterile broth as the negative control.

### 3.8. Statistical Analysis

The inhibition concentration at 50% inhibition (IC_50_) was the parameter used to compare the antibacterial activity. A lower IC_50_ means better antibacterial activity. All of the tests were performed in triplicate and the results were expressed as the mean ± standard deviation. The percent of inhibition of antibacterial activity of the protein was calculated based on: % Inhibition = (A control − A sample)/A control × 100.

### 3.9. Two-Dimensional Electrophoresis

Two-dimensional (2-D) electrophoresis, one the most important tool in proteomics, was performed in accordance with the method reported in [29]. The sample was dissolved in 120 µL of rehydration buffer, loaded onto the 17 cm IEF strip 3–10 pH Liner, (Biorad, Hercules, California, CA, USA), and retained for iso-electric focusing. Next, the strips were equilibrated in equilibrium buffer after finishing the IEF run and the second dimension was run on 12% SDS PAGE.

## 4. Conclusions

Over a long period of time, *Foeniculum vulgare* seeds have shown promise in traditional medicine for the treatment of a wide range of diseases. From our results, 14 spots from the 2D gel were eluted and further subjected to mass spectrometry studies, undergoing more proteomic analyses and the isolation of small peptides from *Foeniculum vulgare*. Many studies have reported that this plant has numerous in vivo and in vitro pharmacological assets that can be used in various aspects of prognosis. Based on the findings of the wide usage in treatment of various ailments and day-to-day life, *Foeniculum vulgare* seeds were used in the present research and have established the promising antibacterial effects of these seeds, which require further proteomic research to authenticate the role of anticipated proteins.

## Figures and Tables

**Table 1:** Protein concentration (μg/mL) in *Foeniculum vulgare* Mill. extract by different buffers after dialysis.

S. No	Plant	Sodium Phosphate Citrate Buffer (pH-7.2) (μg/mL)	CTAB Buffer (pH-6.0) (μg/mL)	Sodium Acetate Buffer (pH-6.5) (μg/mL)
1.	*Foeniculum vulgare* Mill.	80	80	140

**Table 2:** Diameter of the zone of inhibition of sodium acetate buffer, pH-6.5, extracts after dialysis.

S. No	S1	Chl (25 mcg/mL)	Cipro (100 mcg/mL)
*S. aureus*	12	21	16
*E. coli*	11	8	14
*P. aeruginosa*	12.5	8	12
*P. vulgaris*	12	8	14

(S1) *Foeniculum vulgare* Mill., (Chl) Chloramphenicol and (Cipro) Ciprofloxacin. *Staphylococcus aureus* (*S. aureus*), *Escherichia coli* (*E. coli*), *Pseudomonas aeruginosa* (*P. aeruginosa*), and *Proteus vulgaris* (*P. vulgaris*).

**Table 3:** Comparison of the protein concentration (mcg/mL) after ion-exchange chromatography.

S. No	Elute 1	Elute 2	Elute 3	Elute 4
*Foeniculum vulgare* Mill.	120	120	110	100

**Table 4:** Antibacterial activity of ion exchange chromatography elutes from *Foeniculum vulgare*, diameter of the zone of inhibition (mm), and IC_50_ values (mcg/mL).

S. No	Elute 1		Elute 2		Elute 3		Elute 4		Cipro (100 mcg)	
	ZOI	IC_50_ Value	ZOI	IC_50_ Value	ZOI	IC_50_ Value	ZOI	IC_50_ Value	ZOI	IC_50_ Value
*S. aureus*	10	27.64	8	25.91	8	21.27	12	20.8	16	160.529
*E. coli*	13	67.56	12	64.12	6	60.52	8	41.06	14	92.489
*P. aeruginosa*	5	28.01	4	68.33	16	25.02	10	26.67	12	144.634
*P. vulgaris*	12	59.68	13	57.83	6	41.25	7	35.67	14	72.685

**Table 5:** ANOVA analysis of IC_50_ values compared to different strains.

Bacterial Strains	Elute 1	Elute 2	Elute 3	Elute 4	Cipro	*t* Value	*p*-Value *
	IC_50_	IC_50_	IC_50_	IC_50_	IC_50_		
*S. aureus*	27.64	25.91	21.27	20.8	160.529	6.2	0.025
*E. coli*	67.56	64.12	60.52	41.06	92.489	2.39	0.139
*P. aeruginosa*	28.01	68.33	25.02	26.67	144.634	3.4	0.077
*P. vulgaris*	59.68	57.83	41.25	35.67	72.685	6.5	0.023

* One-way ANOVA test. *p* < 0.05.
